# Reconstruction of endosomal organization and function by a combination of ODE and agent-based modeling strategies

**DOI:** 10.1186/s13062-018-0227-4

**Published:** 2018-11-23

**Authors:** Luis S. Mayorga, Ignacio Cebrian, Meghna Verma, Stefan Hoops, Josep Bassaganya-Riera

**Affiliations:** 1 Facultad de Ciencias Médicas, Facultad de Ciencias Exactas y Naturales, IHEM (Universidad Nacional de Cuyo, CONICET), Casilla de Correo 56, 5500 Mendoza, Argentina; 2 Nutritional Immunology and Molecular Medicine Laboratory, Biocomplexity Institute, Virginia Tech, Blacksburg, VA USA; 3 Graduate Program in Translational Biology, Medicine and Health, Virginia Tech, Blacksburg, VA USA; 4 Biocomplexity Institute and Initiative University of Virginia, 995 Research Park Boulevard, Charlottesville, VA 22911 USA

**Keywords:** Intracellular transport, Agent-based modeling, Differential equation modeling, Endocytic pathway, Lipid metabolism, Rab domains

## Abstract

**Background:**

Reproducing cell processes using an in silico system is an essential tool for understanding the underlying mechanisms and emergent properties of this extraordinary complex biological machine. However, computational models are seldom applied in the field of intracellular trafficking. In a cell, numerous molecular interactions occur on the surface or in the interior of membrane-bound compartments that continually change position and undergo dynamic processes of fusion and fission. At present, the available simulation tools are not suitable to develop models that incorporate the dynamic evolution of the cell organelles.

**Results:**

We developed a modeling platform combining Repast (Agent-Based Modeling, ABM) and COPASI (Differential Equations, ODE) that can be used to reproduce complex networks of molecular interactions. These interactions occur in dynamic cell organelles that change position and composition over the course of time. These two modeling strategies are fundamentally different and comprise of complementary capabilities. The ODEs can easily model the networks of molecular interactions, signaling cascades, and complex metabolic reactions. On the other hand, ABM software is especially suited to simulate the movement, interaction, fusion, and fission of dynamic organelles. We used the combined ABM-ODE platform to simulate the transport of soluble and membrane-associated cargoes that move along an endocytic route composed of early, sorting, recycling and late endosomes. We showed that complex processes that strongly depend on transport can be modeled. As an example, the hydrolysis of a GM2-like glycolipid was programmed by adding a trans-Golgi network compartment, lysosomal enzyme trafficking, endosomal acidification, and cholesterol processing to the simulation model.

**Conclusions:**

The model captures the highly dynamic nature of cell compartments that fuse and divide, creating different conditions for each organelle. We expect that this modeling strategy will be useful to understand the logic underlying the organization and function of the endomembrane system.

**Reviewers:**

This article was reviewed by Drs. Rafael Fernández-Chacón, James Faeder, and Thomas Simmen.

**Electronic supplementary material:**

The online version of this article (10.1186/s13062-018-0227-4) contains supplementary material, which is available to authorized users.

## Background

**Fig. 1:**
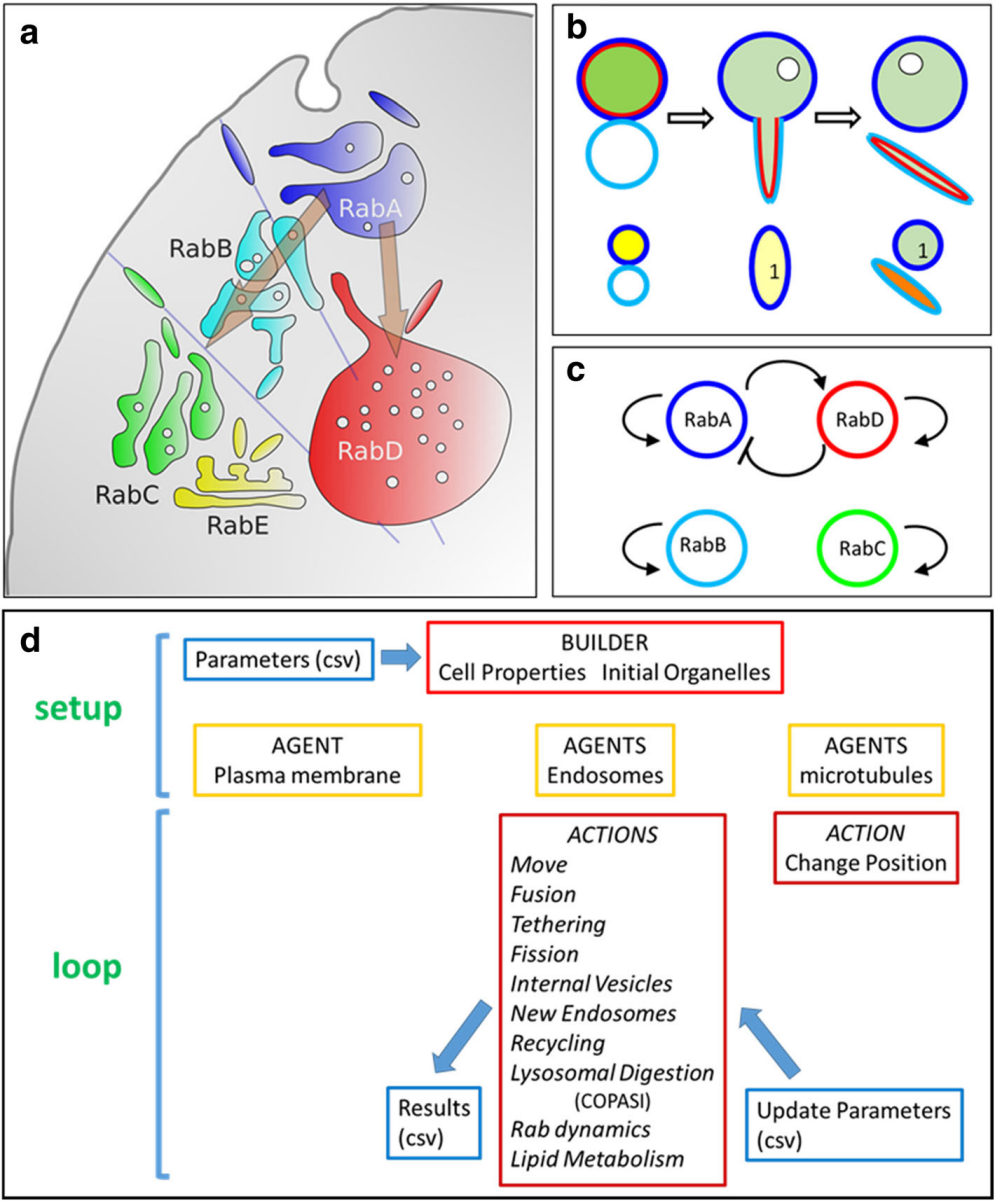
Model description. **a** Informal model for transport along the endocytic route. Different Rab domains characterize four organelles: early endosomes (RabA), sorting endosomes (RabB), recycling endosomes (RabC), and late endosomes (RabD). A trans-Golgi network (TGN) is included as a RabE organelle for modeling transport of lysosomal enzymes (Figs. 7, 8 and 9). The straight lines represent microtubules that orientate the organelle movements. The brown arrows represent the movement of cargoes to recycling endosomes and to late endosomes/lysosomes. **b** Fusion and asymmetric fission. A RabA (blue perimeter) endosome carrying a membrane-bound cargo (red internal circle) and a soluble cargo (green content) fuses with an empty RabB (cyan) endosome. The hybrid vesicle has enough membrane to embrace the volume of the original endosomes, to form an internal vesicle, and to grow a tubule. When the tubule is separated from the round organelle, it carried the RabB domain, the red cargo and an amount of soluble cargo proportional to its volume. At the bottom, the way these organelles are representation in the model. The red and green contents are shown in the interior of the RabA organelle (yellow content). The color of the endosomes corresponds to the prevailing Rab in each organelle. After fusion, the resulting organelle is shown as an ellipse representing the ellipsoid with a size, and area/volume ration of the fused organelle. The color is set to blue because it is the prevailing Rab domain in the organelle. The interior is set to light yellow, because of the dilution of the green and red contents, and a “1” character is plotted indicating that the organelle carries an internal vesicle. After fission, the round organelle is represented as a circle with a blue perimeter, a “1” character (the internal vesicle), and a light green internal color, showing that part of the soluble content and all the membrane cargo went to the tubule. The tubule is represented by an elongated ellipse, with a cyan color corresponding to the RabB domain, and an orange interior representing the red content and part of the green content. **c** Informal representation of the Rab dynamics operating in the model (cut-out switch, [22]). Each Rab domain stimulates its own activation. In addition, RabA promotes RabD activation and RabD inhibits RabA activation. This dynamic was programmed in COPASI and the species, reactions, kinetic functions, and parameters are shown in Table 3. **d** Diagram of the simulation. Initially, Repast builds a set of organelles specified by parameters and characteristics read from a file (Additional file 1). The program then initiates an iterative loop where each agent is interrogated about performing or not a series of actions and changing its properties accordingly. The simulation records the evolution of the system and receives instructions from the user by periodically writing and reading external files (Additional file 2)

Depict the fact that we have a detailed catalog of factors and processes necessary for intracellular transport, our understanding of the underlying molecular mechanism is still fragmentary and qualitative. Generally, models (following Gunawardena’s definition for models: “some form of symbolic representation of our assumptions about reality” [1]) related to the movement of compounds along the endocytic pathway are informal (“one in which the symbols are mental, verbal, or pictorial, perhaps a scrawl of blobs and arrows on the whiteboard”; see Fig. 1a for an example). These informal models are powerful tools to envision potential molecular mechanisms, general cell strategies, and qualitative behaviors. However, they are difficult to validate (or falsify) because they are so vague that several alternative interpretations exists. For example, the brown arrows in Fig. 1a symbolizes the recycling and degradative pathways of an internalized molecule; however, they can also be interpreted as time and/or space fluxes. Moreover, it is not clear whether the depicted compartments remain stable or evolve as the cargoes are traveling along these two pathways. In brief, it is challenging to propose or construct models that describe the flux of cargoes through dynamic structures that continuously change shape, position, and composition.

Mathematical models have proven to be useful tools for understanding the underlying mechanisms and emergent properties in cell biology. Excellent tools are available to represent and model complex biological pathways [2, 3]. However, in the cell, many molecular interactions occur on the surface or the interior of membrane-bound compartments that continuously change position and undergo the dynamic processes of fusion and fission. At present, the available simulation tools fail to incorporate the dynamic evolution of organelles. In particular, it is troublesome to simulate the trafficking of macromolecules along the endocytic and secretory pathways [4]. In this scenario, our aim is to develop a platform for intracellular transport modeling that combines Agent-Based Modeling (ABM) and Ordinary Differential Equations (ODE) that are able to handle the dynamic nature of organelles along with the complex network of molecular interactions that occur in individual structures. In particular, we focused on the endocytic route.

Endocytosis has a central role in the physiology of the cell. It is required for the uptake of macromolecules to be processed in lysosomes, the internalization and recycling of receptors involved in several cell signaling cascades, and a myriad of diverse functions that occur in these membrane-bound organelles. By functional and morphological criteria, the endocytic route is divided into several compartments. Early endosomes receive the material endocytosed by different mechanisms (clathrin-dependent and independent endocytosis, macropinocytosis, phagocytosis, to name a few). From this early compartment, the compounds can directly recycle back to the plasma membrane (early recycling) or can be transported to the sorting endosomes where the molecules are directed to their specific intracellular destinations. Several compounds are directed to the endosomal recycling center, located near the nucleus, and responsible for the slow recycling to the cell surface. If the endocytosed materials are not sorted out from early endosomes, their final destination is the late endosomal/lysosomal compartment triggered by the maturation of early to late endosomes [5]. The presence of hydrolytic enzymes and acidic environment in the lysosomes promote the digestion of most of the biological macromolecules.

To develop a formal model of the endocytic pathway, we utilized the concept that endosomes are characterized by their limiting membranes. Membrane domains are relatively stable structures that do not necessarily mix when residing side by side in the same organelle [6, 7]. Membrane domains can thus be considered as the key building blocks of the endomembrane system of the cell. Transport can be envisioned as the change in the limiting membrane of organelles carrying a cargo by means of mixing (fusion of organelles) and sorting (budding and fission of vesicles and tubules) processes. In addition, complex networks of molecular interactions change the protein and lipid composition of the limiting membrane of the organelles [8, 9]. Soluble cargoes that do not interact with the membranes are transported in the lumen of the organelles. During the budding of vesicles and tubules, these cargoes are partitioned in volumes proportional to those of the splitting structures; hence, very little is incorporated in the newly-formed membrane-rich structures whereas a large proportion is retained in the remaining round organelle that carries most of the total volume. Since tubules are key components of the recycling pathways, most soluble cargoes are delivered to the lysosomes [10]. On the contrary, membrane-bound factors travel through the endomembrane system according to their affinity for membrane domains that are sorted out during the budding of vesicles or tubules. Membrane association depends on specific interactions of tags coded in the cargo molecule with one or more of the large set of adaptor proteins present in the cell [11, 12]. In addition, lipids on the membranes undergo lateral phase separations, and membrane-anchored factors can also be recruited to specific lipids domains [13]. Depending on their protein and lipid composition, different membrane domains bind membrane-deforming protein complexes, such as COPs, clathrin, sorting nexins, ESCORT, etc. [14–17]. The deformations lead to the sorting of membrane domains by budding of tubules or vesicles, or the isolation in intraluminal vesicles that are now separated from the membrane of the original organelle, carrying a subset of cargo molecules. Membrane domains depend on the coordinated activity of multiple factors, including several members of the Rab family [7, 18]. Perturbation of Rab functions, dramatically alters the intracellular transport of most of the cargoes [19, 20]. Rabs are small GTPases that act as molecular “on/off” switches, cycling between inactive (GDP-bound) and active (GTP-bound) states. They can be organized in cascades adding flexibility and regulation to the identity of membrane domains [5, 6, 21]. Furthermore, Rab proteins localize to specific membrane-bound compartments; hence, they can be used to identify specific membrane domains.

The simulation implemented using the modeling platform reproduced many aspects of the endocytic transport. Enzymes, molecular pumps, structural proteins, and lipids can be included in the simulation to study complex signaling and metabolic networks. The model captures the highly dynamic nature of cellular structures that fuse and divide, creating particular conditions for molecular interactions that are different for each organelle. We expect that this combination of modeling strategies will be useful for cell biologists to generate formal models that can be used to unveil the essential mechanisms underlying the highly efficient transport processes observed in living cells.

## Results

### Brief model description

In this section, we provide a simplified and intuitive description of the model. More details can be found in Methods and in the notes included in the Repast and COPASI code that are freely accessible at the Git repository https://github.com/ihem-institute/immunity/tree/LipidMetabolism.

Organelles carrying four different membrane domains were modeled in Repast (ABM platform). For simplicity, we can think of organelles limited by RabA as early endosomes, by RabB as sorting endosomes, by RabC as recycling endosomes, and by RabD as late endosomes/lysosomes (Fig. 1a). The organelles, carrying membrane and soluble content were allowed to move, interact and change their shape with time. To setup the simulation, the Repast Builder generates a set of initial organelles according to the parameters and characteristics selected by the user (Fig. 1d and Additional file 1). Repast then runs a loop of actions for all the agents in the model as listed in Fig. 1d and described in Methods. The user can control the model by updating parameters or pausing the simulation. The model continuously generates records of the evolution of the system as requested by the user (example in Additional files 2 and 3).

#### Shape and movement

**Fig. 2:**
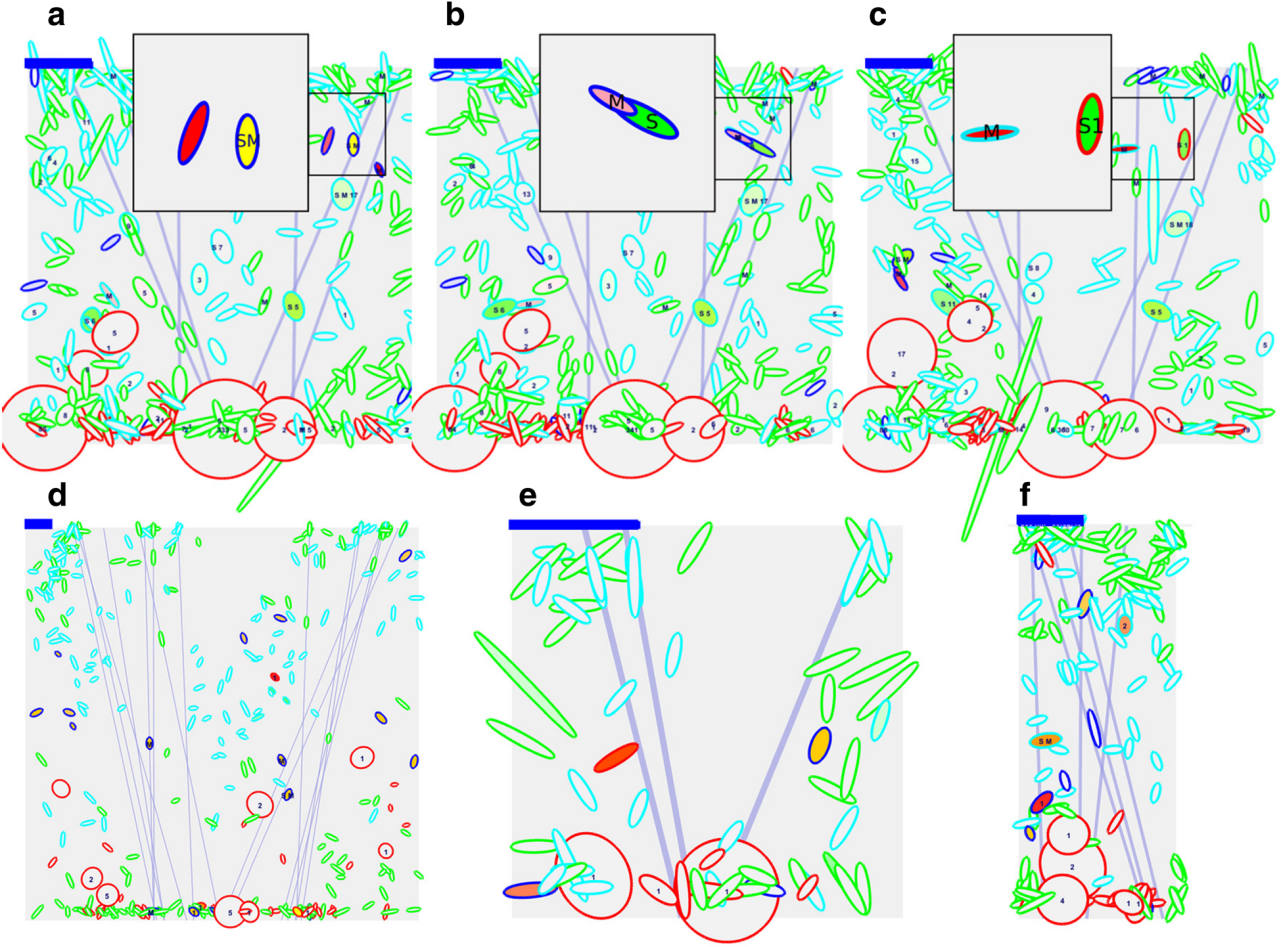
Snapshots of simulations plotted by Repast. **a**-**c** The same simulation is shown at three different times. **a** The inset shows an early endosome (blue perimeter), carrying Tf (red) and dextran (green) cargoes (red + green = yellow content), and also a soluble (S) and a membrane (M) markers. Nearby another early endosome containing just Tf (red content) is present. **b** After fusion and fission, the membrane marker is segregated to an endosome containing mostly Tf, and the soluble marker to an early endosome containing mostly dextran (inset). **c** Finally, the endosome containing the soluble marker (S) and dextran matured to a RabD structure (red perimeter) containing an internal vesicle (“1” character). The endosome carrying the membrane marker (M), after fusion and fission is now a RabB endosome (cyan perimeter) containing Tf (inset). **d** A larger world of 9 × 9 μm with much more organelles. **e** A reduced world of 2.25 X 2.25 μm. **f** A rectangular world of 2.25 X 4.5 μm. The blue bar at the upper left corner of the worlds corresponds to 0.5 μm

Endosomes in the model have a membrane area that embraces a luminal volume and are represented by ellipsoids (spheres for round structures and elongated ellipsoids for non-round endosomes, depending on the volume/area ratio; Figs. 1b, 2, and movie in Additional file 4). The size of the organelles is represented in a nm scale. The endosomes are allowed to move randomly except when near a microtubule (MT, light blue straight lines in the model, Figs. 1a, 2, and movie in Additional file 4). Round and tubular organelles on MT, move to the surface or to the center of the cell according to their membrane domains at a speed of 1 μm/sec.

#### Fusion, fission, and luminal vesicles

Two closely spaced endosomes with compatible membrane domains are allowed to fuse and form a single organelle. This organelle carries all the membrane and soluble components of the original endosomes. The endosomal compatibilities are listed in Table 1. Endosomes with enough membrane can also split, and form tubules (40 nm diameter) carrying a single membrane domain. The soluble content is distributed proportionally to the volume and the membrane components proportionally to the area of the newly formed structures. The membrane-associated cargoes may have affinities for some specific membrane domains, and will partition during fission according to these affinities. Endosomes can also form internal vesicles, thereby decreasing their limiting area and increasing their volume (the number of internal vesicles is shown as a number inside each endosome, Figs. 1b and 2). Note that the area, volume, Rab, membrane, and soluble contents were preserved during fusion and fission events.

**Table 1:** Parameters used to characterize the organelles simulated in the model

	Fusion and tethering probability					Recycling probability	Tubulation tendency	Migration on MT	Rab switch
	RabA	RabB	RabC	RabD	RabE				
RabA	1	0.5	0.0001	0.001	0.001	0.1	0.8	1	→ RabD
RabB	0.5	1	0.3	0.03	0.01	0	1	1	–
RabC	0.0001	0.3	1	0.0001	0.0001	0.3	1	0.5	–
RabD	0.001	0.03	0.0001	0.1	0.001	0	0	− 1	–
RabE	0.001	0.01	0.0001	0.001	1	0	1	−1	–

To model a particular intracellular transport process, the membrane domains surrounding the organelles must be enumerated and characterized. For a standard endocytic route, we have incorporated early (RabA), sorting (RabB), recycling (RabC), and late (RabD) endosomal domains. A TGN domain was also included (RabE). The fusion probabilities were selected according to what is generally accepted in the intracellular transport field (e.g., early and sorting endosomes mix frequently, whereas early and late endosomes seldom fuse). Recycling probabilities were assigned with the same criteria. Tubulation tendency was low for domains surrounding large round organelles (such as late endosomes/lysosomes). Migration on MT was assigned according to the reported position of the organelles in the cell (late endosomes, TGN, and recycling endosomes proximal to the nucleus, and early and sorting endosomes near the plasma membrane). In the pathway simulated, the only switch included was RabA → RabD. Rab cascades like this must be programmed in COPASI, by changing the set of enhancers and inhibitors for the corresponding reactions in the rabs_convertion COPASI file (Additional file 5)

#### New endosomes

When new RabA organelles were formed, they were modeled as an endocytic event, budding from the cell surface and carrying soluble and membrane-bound components. 

#### Recycling

When endosomes carrying RabA or RabC membrane domains were proximal to the plasma membrane, they could release their membrane and soluble content (recycled material), that was incorporated into the plasma membrane or released to the medium.

#### Lysosomal digestion

Endosomes carrying RabD domains digest part of their content and membrane.

#### Rab dynamics

We generated a set of ODE in COPASI to model the self-preserving behavior of membrane domains and a Rab switch homologous to the Rab5/Rab7 conversion to regulate the transformation of early (RabA) to late (RabD) endosomes (Fig. 1c, Table 3, and Additional file 5) [22]. COPASI and Repast communication was achieved using a previously published method [23]. Hence, in the simulations, Rab dynamics was controlled by differential equations (COPASI) and the rest of the model by ABM (Repast).

A global analysis of the endocytic route proposed shows four stable compartments that communicate with each other by fusion and fission events (according to membrane domain compatibilities shown in Table 1. Notice that homotypic fusion has the highest probability for all compartments. The exchange of material during fusion/fission processed is in principle bidirectional. However, the flux can be orientated according to how the molecules distribute during fission events. On top of this organization, two irreversible steps strongly influence transport: the maturation of early to late endosomes and the recycling to the plasma membrane. In the present model, only the content is recycled; hence recycling endosome domains are preserved. In contrast, maturation caused the consumption of early endosomes and the building up of late endosomes. To compensate for this, new early endosomes are constantly formed and lysosomal digestion consumes RabD domains.

### Model characterization

In ABM models, during a tick interval, all agents are interrogated about performing or not a set of actions (Fig. 1d and Methods). The tick duration was calibrated with the fastest process in the model (movement of organelles on MT; 1 tick = 0.06 s). The model was stable for more than 180.000 ABM ticks corresponding to about 3 h in “cellular” time. The characteristics of the resulting organelles correspond to a rather standard endocytic pathway with organelles moving in and out the “cell” surface by random movements or along microtubules (Fig. 2 and movie in Additional file 4). Large round-shaped structures accumulated opposite to the plasma membrane (in the nucleus side). Fusion and fission of the organelles triggered the mixing and segregation of membrane domains, while the RabA/RabD switch guaranteed the eventual maturation of early to late structures. Events of fusion, fission, maturation and cargo transfer are shown in the inset of Fig. 2a-c and explained in the figure’s legend.

**Fig. 3:**
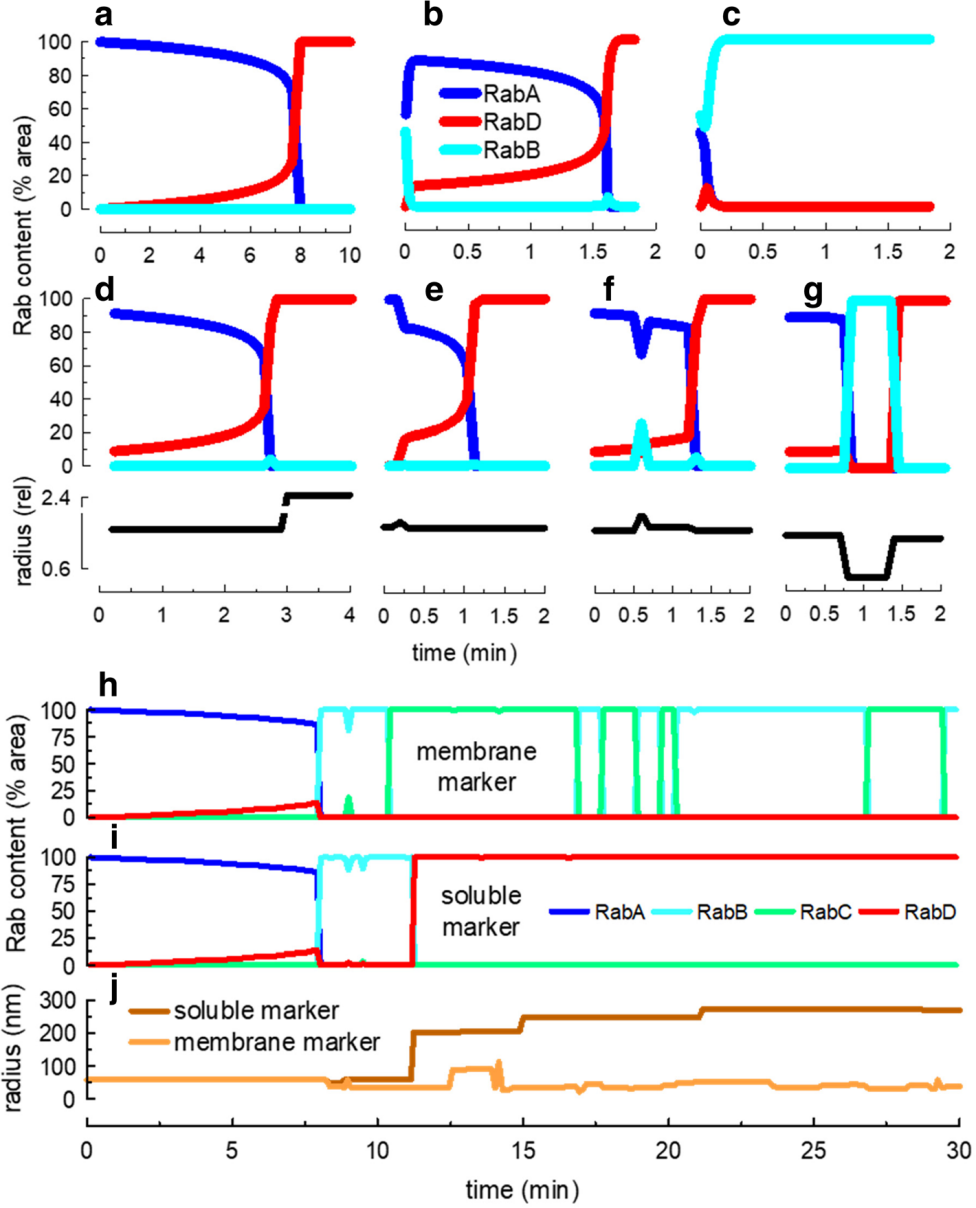
Rab dynamics in endosomes. **a** Maturation of an isolated RabA endosome to a RabD endosome. **b** Same as **a**, but the initial Rab content was 55% RabA, 45% RabB. **c** Here, the initial Rab content was 45% RabA, 55% RabB. Notice that the endosome evolved into a pure RabB structure. **d**-**g** Several RabA, RabB, RabC and RabD endosomes were created and allowed to fuse and divide. A membrane marker was initially loaded in a RabA endosome. With time, the endosomes carrying the markers changed their membrane composition by means of RabA maturation and a combination of fusion and fission. The size (relative to the initial value) of the endosomes carrying the markers is plotted underneath each panel. **h**-**j**. A soluble and a membrane marker were loaded in a RabA endosome. The endosomes containing the markers were followed during 30 min. In **h** the membrane marker was initially in a RabA endosome; afterwards, it moved back and forward between RabB and RabC endosomes until it was recycled. **i** The soluble marker suffered the same fate than the membrane marker until it was transferred first to a RabB endosome and then to a RabD endosome by means of several fusion and fission events. **j** The size of the endosomes carrying the soluble and membrane markers is shown. The size is expressed as the radius of a sphere having the volume of the endosome

If a single endosome was generated, a RabA organelle matured to RabD according to the molecular switch modeled by differential equations in COPASI (Fig. 1c and Table 3). With time, RabD was incorporated into the organelle and finally the switch occurred (Fig. 3a). When other Rabs were also present, the process was more complex and the prevailing Rab tended to displace less abundant Rabs. Fig. 3b shows what happened with an organelle that initially had 45% of its surface covered with RabB and 55% with RabA. In this condition, RabB disappeared and RabA covered the endosome to, later on, switched to RabD. However, notice that the switch kinetics was altered by the presence of RabB. On the contrary, if 55% of the organelle was initially covered with RabB, the endosome rapidly evolved to an all-RabB structure (Fig. 3c).

The scenario was far more complex when other endosomes were included in the model. The molecular switch was profoundly affected when a new membrane domain was incorporated by fusion with other endosomes or when fission segregated a complete membrane domain from the organelle. Examples of RabA/RabD switches are shown in Fig. 3 d to g. In Fig. 3d, a fusion occurred just after the switch, causing a large increase in the endosome size (size are shown as relative to the initial endosome radius underneath the corresponding switch process). In Fig. 3e, there was a fusion (size increase) and fission (size decreased) after which, the endosome matured to a RabD structure. In Fig. 3f, there were several fusion and fission events that profoundly affected the switch kinetics. Finally, in Fig. 3g, the endosome was first converted to a RabB structure and then to a RabD organelle by fusion and fission processes.

The behavior of this complex scenario can be analyzed in different ways. For example, although in Fig. 3a to c it is clear that a single endosome was analyzed, it is not obvious which endosome is followed in Fig. 3d to g. This is due to the occurrence of fission events that make it unclear which structure is described. To avoid this ambiguity, markers, modeled as soluble or membrane-associated cargoes, were loaded in the endosomes. The analysis of the organelles that contain a specific marker during the course of the simulation provided meaningful information about the transport events for a specific cargo. In Fig. 3d to g, a membrane marker was included and the Rab composition of the endosome carrying the marker was followed along the time. Fig. 3h and i, show the characteristics of organelles carrying a membrane and a soluble cargo along the course of the 30 min simulation. Both markers were initially loaded in the same RabA structure. Notice that both sensed the same initial steps of a RabA/RabD switch, but before maturation was completed, fusion and fission events transformed the organelle in a RabB structure, from where the fate of the two markers diverged. The membrane marker was segregated in a RabC organelle and for a while kept moving between endosomes carrying RabB and RabC domains, until it was recycled to the plasma membrane from a RabC endosome. Notice the changes in the organelle size that oscillated between 40 and 100 nm radius (Fig. 3j). The soluble marker instead, was transported from a RabB endosome to a large RabD organelle that kept growing by fusion with other RabD structures (Fig. 3j).

**Fig. 4:**
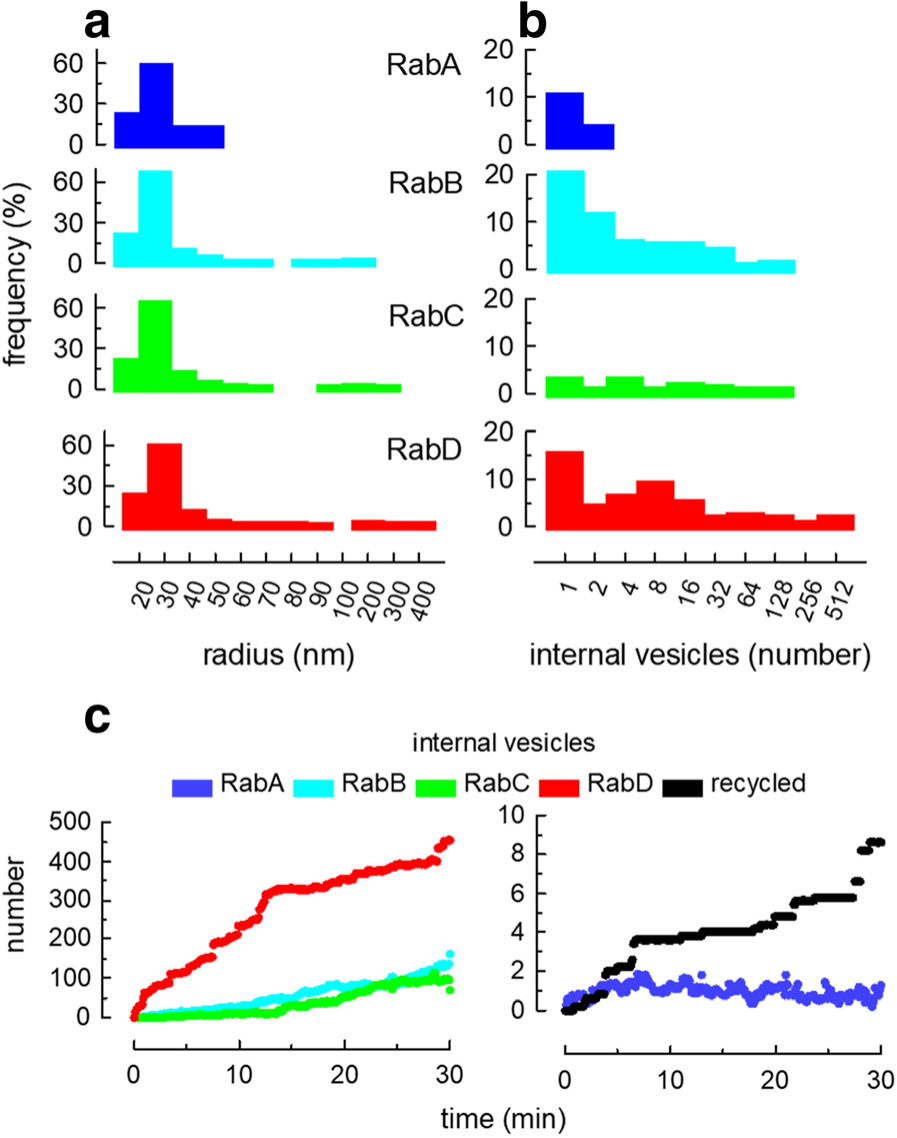
Characterization of organelles in the simulation. **a** Size distribution in a population of endosomes after 30 min of simulation. The endosomes were classified according to their prevailing Rab domain and the size was calculated as the radius of a sphere having the same volume than the endosome. Notice that RabA structures were small and that the largest endosomes carried RabD domains. **b** Same as in **a**, but the frequency of endosomes carrying different numbers of internal vesicle is plotted. For panels **a** and **b**, the endosomes of 5 simulations were used. **c** A different manner to assess the distribution of internal vesicles among organelles. For this calculation, all internal vesicles contained in RabA endosomes were added and plotted at different time of simulation (see Methods). Same calculations were done for each Rab domain. The left panel shows the internal vesicles accumulated in RabD, RabB and RabC structures. The panel at the right, in an expanded scale, shows the internal vesicles in RabA endosomes and the number of vesicles released at the plasma membrane. The values for this figure correspond to the average of 5 identical simulations

A different way to analyze the simulation is to take all the organelles at a given time and classify the structures according to the prevailing Rab domain as early, sorting, recycling or late endosomes and to analyze the characteristics of these structures. Histograms in Fig. 4a and b show the size and internal vesicle content distribution of all organelles classified according to their prevailing Rab domain after 30 min of simulation. Notice that all the organelles have some small endosomes (radius in the 20–40 nm range); however, large structures (radius > 300 nm) were more frequently observed in late endosomes (RabD) and were absent in the early endosomes (RabA). The size of the largest endosomes carrying RabB or RabC domains had an intermedia radius in the range of 100–200 nm (Fig. 4a). Internal vesicles were observed in all endosome types, but they were more frequently seen in RabB and RabD structures (Fig. 4b).

Another analysis that we performed includes the calculation of the amount of a cargo associated with the different compartments at each time point of the simulation (see Methods). For example, the total number of internal vesicles in the simulation can be classified as being associated to either RabA, RabB, RabC or RabD structures (Fig. 4c). Notice that most of the vesicles accumulated in the RabD endosomes. About 500 internal vesicles were observed in the RabD structures at time 30 min, whereas, RabB and RabC had about 100 vesicles (Fig. 4c, left panel) and RabA had only 1 vesicle (Fig. 4c, right panel). In the expanded scale shown at the right, it can be seen that some internal vesicles recycled to the plasma membrane, a process that mimics the release of exosomes from multivesicular bodies (recycled, Fig. 4c, right panel).

### Transport of soluble and membrane-bound cargoes

In the complex scenario simulated, transport of a cargo molecule is profoundly affected by its behavior during fission events. Like proteins carrying a specific tag in their sequence, cargo moved in this artificial endocytic route depending on their affinity for membrane domains that conditioned their segregation in one or other structure during the budding and fission of tubules. On top of these processes, the maturation of early endosomes will transport to late endosomes all cargoes that are not sorted out to different organelles. Soluble cargoes are not recognized by membranes and accumulated in the vesicular (large volume) structure that remains after the formation of tubules. Moreover, large cargoes (larger than 40 nm diameter, like intraluminal vesicles) are excluded from tubules. These cargoes are prompted to be finally delivered to large late endosomes (Fig. 4c).

**Fig. 5:**
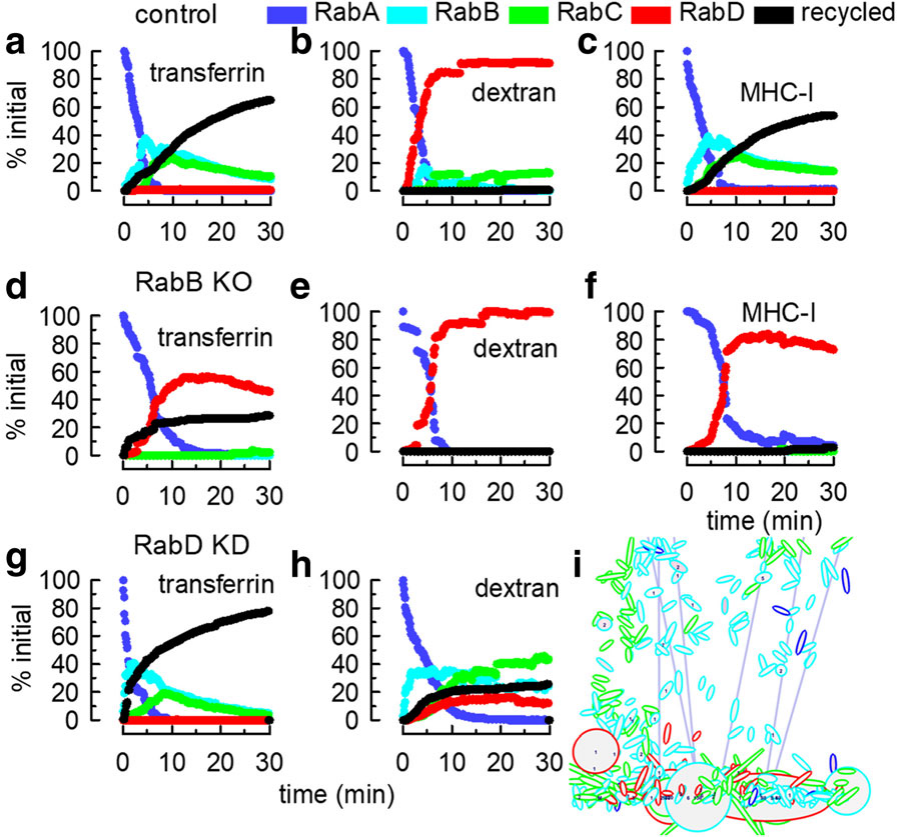
Pulse/chase of a soluble (dextran-like) and two membrane-bound (Tf-like and MHC-I-like) cargoes. The molecules were loaded in RabA structures at time 0 of simulation. **a**-**c** Association of cargoes with different Rab domains and recycling to the plasma membrane during different chase times. Notice that the membrane markers efficiently recycled to the plasma membrane whereas the soluble marker accumulated in RabD endosomes. **d**-**f** Same as in **a**-**c**, but when RabB structures were not included in the simulation. Notice that when the RabB compartment was absent, the recycling of Tf was strongly inhibited and that of MHC-I was abolished. In contrast, transport of dextran to late endosomes was only slightly delayed. **g** and **h** Same as in A-C (MHC-I not shown), but when cytosolic RabD was set to zero. Notice that when the RabA→RabD switch was inhibited, the recycling of Tf was not impaired, whereas dextran transport to RabD endosomes was diminished and this soluble cargo accumulated in RabB and RabC structures. **i** Snapshot of a simulation without cytosolic RabD. Notice the presence of large RabB endosomes (cyan). The values for this figure correspond to the average of 8–10 identical simulations

To analyze the behavior of different cargoes, a soluble (dextran-like) and two membrane-bound cargoes were loaded into early endosomes (RabA) and the model was allowed to evolve for 30 min. The two membrane proteins simulated included: i) a transferrin-like cargo (Tf) that could recycle from early endosomes and recycling endosomes, and ii) a class I major histocompatibility complex -like cargo (MHC-I) that needed to be sorted to RabB structures before being recycled from RabC endosomes. Both proteins were provided with membrane affinities for RabB and RabC domains (Table 2). This means that they were sorted during fission in structures rich in these Rab domains. To analyze the movement of the cargoes, the percentage of each molecule in organelles with different membrane domains was calculated and plotted in Fig. 5. The data showed the average of 8–10 simulations and reproduced the well-characterized kinetics of pulse-chase experiments for these ligands. A clear difference was observed between the two membrane proteins Tf and MHC-I, which recycled to the plasma membrane, and dextran that accumulated in late endosomes (Fig. 5a to c). The possibility of recycling from early endosomes made the kinetics of Tf recycling more efficient than that of MHC-I. Experimental data have shown that perturbation of sorting endosomes (for example, knocking down Rab22a) profoundly affect the recycling of MHC-I molecules [24, 25] and also, although to a lesser extent, that of Tf [26]. In contrast, transport of soluble markers was only marginally altered [27]. In the simulation, when we knocked out RabB structures, these observations were reproduced fairly well. Tf recycling was strongly diminished, but not abolished whereas the recycling of MHC-I was completely blocked. Transport of dextran to late endosomes was only slightly delayed (Fig. 5d to f).

**Table 2:** List of molecules included in the simulation and their affinities for Rab domains

Cargo molecules	affinity for the Rab domains				
	RabA	RabB	RabC	RabD	RabE
GM2				1	
M6PR					1
M6PR-HexA					1
HexA	soluble				
cholesterol	1	0.5			
vATPase	0.7	1			
proton	soluble				
Tf		1	1		
MHC-I		1	1		
dextran	soluble				
solubleMarker	soluble				
membraneMarker		1	1		

The molecules that are necessary to model a specific process are enumerated and their affinity for membrane domains specified. For example, to model the hydrolysis of a ganglioside, we included the relevant enzyme, the receptor that transports the enzyme, and cholesterol and protons that modulate the enzyme activity. Other cargo molecules whose transport kinetics are well known, such as Tf, MHC-I, and dextran were added. Soluble and membrane markers are special cargos that are useful to assess the characteristics of the organelles that a selected molecule visit during the simulation. Soluble molecules are easily identified. For a membrane-associated molecule, the affinity for the different domains is inferred from its a priori known destination in a cell. For example, M6PRs are retrieved back to the TGN, so the receptor and the enzyme-bound receptor are given affinity for the TGN domain. Tf and MHC-I are given affinity for RabB and RabC to be sorted out from RabA compartments that eventually mature to late endosomes. Cholesterol is enriched in early endosomal structures and GM2 is sequestered in intraluminal vesicles that accumulate in late endosomes/lysosomes

In a different set of simulations, cytosolic RabD was depleted, preventing the maturation of RabA structures. Since RabD organelles were still present in the model, we homologate this condition to a RabD knockdown. The decreased cytosolic RabD inhibited the accumulation of dextran in late compartments and caused a dramatic increase of the fluid phase marker in sorting and recycling endosomes (Fig. 5h). In contrast, Tf recycling was not impaired. The effects observed closely reproduce what is experimentally observed when Rab7 function is perturbed [28]. The appearance of large sorting and recycling endosomes was evident (compare Fig. 5i with Fig. 2), a phenotype that has been described in cells depleted of Rab7 or expressing a dominant negative mutant of this protein [29].

In all these simulations, the endocytic markers were incorporated in RabA compartments at time zero. In a different protocol, RabA endosomes were empty and uptake was allowed in the 3–6 min time period. Under these conditions, transferrin and dextran were recycled and transported to late endosomes, respectively, with similar kinetics as observed in the time zero protocols (Additional file 6). These results indicate that transport was not strongly affected by the initial conditions of the system. In the figure shown in Additional file 6, six individual runs of the same simulation are shown. Given the stochastic nature of transport, all simulations present particular uptake events and cargo transport along time. However, in all simulations, there was a consistent recycling of transferrin and transport of dextran to late endosomes.

### Exploring the power of adding molecular functionality to the simulation

The simplified endocytic route modeled here and the mechanisms for cargo transport proposed reproduced quite well the behavior of several molecules transported along this pathway. But the real strength of this simulation platform is the possibility of easily adding molecular functionality to the structures. For example, a set of differential equations was modeled in COPASI to simulate endosomal acidification. Each organelle was allowed to pump proton into the lumen by means of a membrane-associated vATPase. Leakage reactions allowing the in and out movement of protons were also included (see Methods, Table 3, and Additional file 7). The vATPase was given low affinity for RabB and RabC domains (0.0), high affinity for RabD structures (1.0) and a moderate affinity for RabA (0.7) (Table 2). With these values, the vATPase was enriched in the early/late endosome path, but some activity leaked to other compartments, a distribution that fairly mimicked what is observed in most cells [30].

**Table 3:** Set of reactions and kinetic functions programmed in COPASI

	Name	Reaction	Inhibitor(s)	Activator(s)
Rab Dynamics				
Kinetic function: $\frac{\mathrm{kS}1}{\left(1+\left(\exp {\left(\mathrm{kS}2-\sum activators\right)}^{\mathrm{kS}3}\right)+\exp \left(-{\left(\mathrm{kS}4-\sum inhibitors\ \right)}^{\mathrm{kS}5}\right)\right)}\ast \prod substrates$ kS1 = 11; kS2 = 0.8 ; kS3 = 2; kS4 = 0.2; kS5 = 13				
	RabA Activation	RabAc + Rab0 → RabAm	RabDm	RabAm
	RabA Inactivation	RabAm → RabAc + Rab0	RabAm	RabDm
	RabB Activation	RabBc + Rab0 → RabBm		RabBm
	RabB Inactivation	RabBm → RabBc + Rab0	RabBm	
	RabC Activation	RabCc + Rab0 → RabCm		RabCm
	RabC Inactivation	RabCm → RabCc + Rab0	RabCm	
	RabD Activation	RabDc + Rab0 → RabDm		RabDm, RabAm
	RabD Inactivation	RabDm → RabDc + Rab0	RabDm, RabAm	
	RabE Activation	RabEc + Rab0 → RabEm		RabEm
	RabE Inactivation	RabEm→RabEc + Rab0	RabEm	
Kinetic function: k*[substrate] k = 0.3				
	rA Influx	RabAcyto → RabAc		
	rA Outflux	RabAc → RabAcyto		
	rB Influx	RabBcyto → RabBc		
	rB Outflux	RabBc → RabBcyto		
	rC Influx	RabCcyto → RabCc		
	rC Outflux	RabCc → RabCcyto		
	rD Influx	RabDcyto → RabDc		
	rD Outflux	RabDc → RabDcyto		
	rE influx	RabEcyto → RabEc		
	rE Outflux	RabEc → RabEcyto		
Endosome Acidification and Lipid Metabolism				
Kinetic Function: k*[substrate] k Proton Pump = 0.0008; k_1_ Proton leak = 0.04; k_-1_ Proton leak = 0.04; k Cholesterol depletion 10				
	Proton Pump	vATPase → proton + vATPase		
	Proton leak	protonCy ←→ proton		
	Chol depletion	cholesterol + RabD → RabD		
Kinetic Function: k ∗ (10^pH − pHlimit^)^enhancer^ ∗ ∏ *substrates* pH = -log[proton]; k, pHlimit and enhancer for each reaction are listed bellow HexaA binding: 1, 6, 1.5; HexA release: 2*10^-4^, 6, -1.5				
	HexA release	M6PR-HexA → HexA + M6PR		
	HexA binding	M6PR + HexA → M6PR-HexA		
Kinetic Function: $\frac{10^{-6}}{\left[\mathrm{cholesterol}\right]+{10}^{-6}}\ast {\left({10}^{\mathrm{pH}-5}\right)}^{-2}\ast \prod substrates$ pH = -log[proton]				
	GM2 hydrolysis	GM2 + HexA → HexA		

Rab dynamics on endosomal membranes was modeled following the scheme shown in Fig. 1c, where each Rab activates its own activation and inhibits its inactivation. RabAc and RabAm refer to the GDP- and GTP-bound forms, respectively (idem for other Rabs). Rab0 refers to a Rab free membrane domain. The RabA → RabD switch was programmed adding RabD as an inhibitor of RabA activation and activator of RabA inactivation. In addition, RabA was added as an activator of RabD activation and inhibitor of RabD inactivation. The kinetic function used was able to handle Rab dynamics in all organelles with only five parameters. The parameters were adjusted to mimic the reported Rab5 → Rab7 conversion kinetics [5]. Membrane-associated Rab-GDPs were allowed to exchange with a cytosolic pool (cyto Rabs). Units in mM and seconds (see Additional file 5 and Additional file 7 for the specific unit of each parameter) For lipid metabolism, the pH of organelles was regulated by a proton pump and a leakage reaction. Cytosolic pH was set to 7. To model the dynamics of HexA association with its receptor (M6PR), a pH-dependent kinetics function was used [51]. Finally, the GM2 hydrolysis was controlled by a pH and cholesterol-dependent kinetic function [33]. All parameters were adjusted to qualitatively reproduce reported experimental observations. Units in mM and seconds (see Additional file 5 and Additional file 7 for the specific unit of each parameter)

**Fig. 6:**
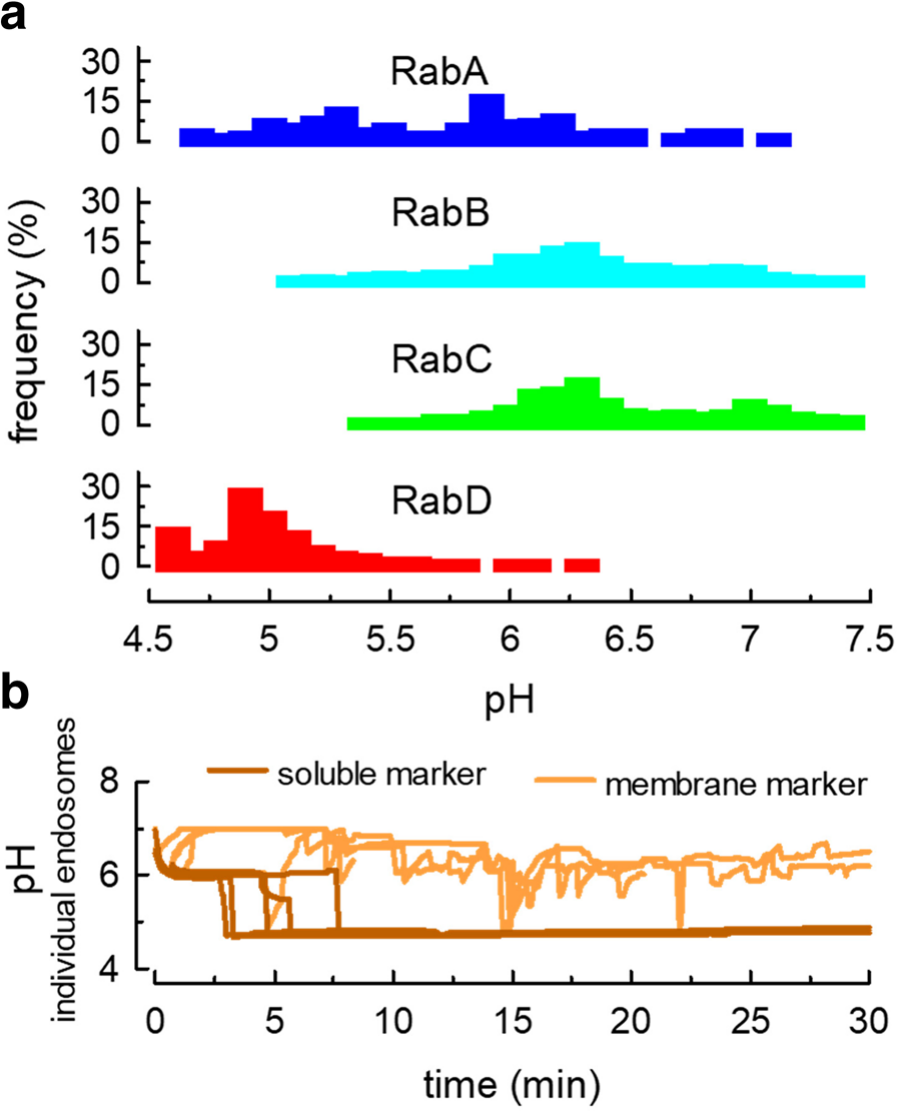
Modeling endosome acidification. A proton pump with affinity for RabA and RabD domains was included in endosomes and the proton concentration was calculated using a set of ODE programmed in COPASI. **a** The endosomes were classified according to the prevailing Rab domain and the pH was calculated from the proton concentration in each structure. The histograms of the pH distribution for each Rab domain were obtained combining the population of endosomes of 5 independent 30 min-runs. **b** Membrane (light brown) and soluble (dark brown) markers were included in RabA endosomes at the beginning of the simulations and the pH sensed by the probes at each time was recorded. The panel shows the pH sensed by 10 markers in 5 independent runs. Three membrane markers recycled to the plasma membrane before the end of the simulations

After a few minutes of simulation, a pH gradient was established in the system. The analysis of this parameter in all endosomes after 30 min of simulation indicated that RabA endosomes had a pH average of 5.7 with a very broad distribution, whereas RabB and RabC were only lightly acidic with pH average of 6.3 and 6.4, respectively (Fig. 6a). On the other hand, RabD organelles had an acidic pH with an average of 5 (Fig. 6a). Following the pH sensed by membrane-associated (MHC-I-like) markers, we observed that as they moved along different organelles, on average, they were exposed to near neutral pH (6.4 ± 0.22, mean ± SD, taking into account the time remaining at each pH, *N* = 10, Fig. 6b). However, the probes transiently visit organelles with low pH (Fig. 6b). On the other hand, soluble markers moved rapidly to acidic organelles rich in vATPase with very few oscillations in the pH (pH ~ 5, Fig. 6b).

**Fig. 7:**
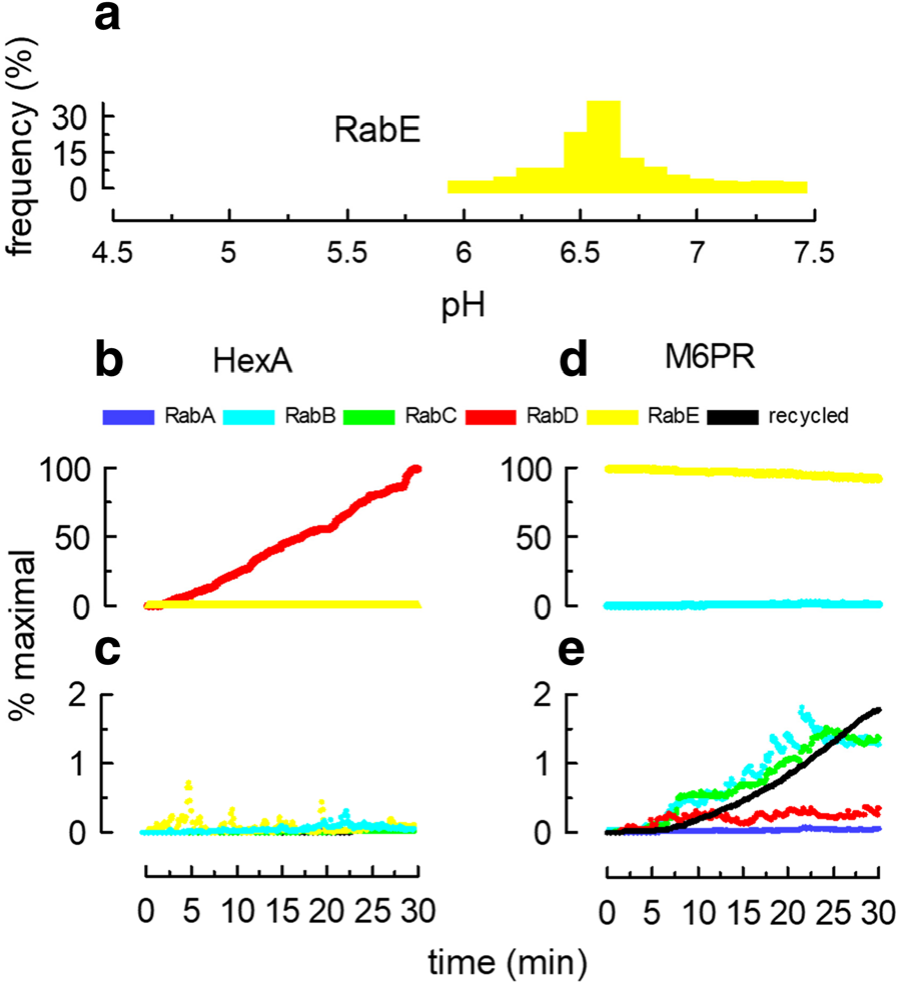
Modeling lysosomal transport from TGN to endosomes. A RabE domain (yellow, shown as a TGN compartment in Fig. 1a) containing a lysosomal enzyme (HexA-like cargo) bound to a membrane-associated transporter (M6PR-like cargo) were included in the simulation. RabE organelles interact with RabB endosomes. The M6PR was given affinity to RabE domains. The reversible dissociation of HexA from M6PR was programmed as a pH-regulated reaction in COPASI (Tables 1, 2, and 3). **a** Histogram of pH for RabE structures from 3 simulations (30 min runs). **b** The amount of soluble HexA (dissociated from M6PR) present in RabD and RabE structures was recorded along the simulation. After dissociation at low pH, HexA behaved as a soluble cargo and accumulated in RabD structures. **c** Same as in **b**, showing the association of the HexA with RabA, RabB, and RabC endosomes or recycled to the plasma membrane. Notice the very low values even for the expanded scale used. **d** The amount of M6PR present in RabB and RabE structures was recorded along the simulation. Most of the receptor was retained in RabE structures. **e** Enlarged scale to show that a small percentage of M6PR was found in RabB and RabC endosomes. The levels were low in RabA and RabD structures. Part of M6PR was transported to the plasma membrane. Data on panel **b** to **e** correspond to the average of 8–10 independent simulations

Endosomal pH is an important property of these organelles and plays a central role in several aspects of the endocytic route. For example, the consensus is that acid hydrolases are delivered to lysosomes by vesicular structures coming from the trans-Golgi network (TGN) that fuse with early/late endosomes [31]. The enzymes, bound to transport proteins (e.g., mannose 6-phosphate receptor, M6PR), are released into the lumen at the acidic pH of endosomes. To simulate this transport in the model, a new organelle carrying a different membrane domain was included (Fig. 1a). This organelle was given a RabE domain that could interact with RabB organelles (Table 1). A lysosomal enzyme (β-hexosaminidase A, HexA) bound to a membrane-associated transporter (M6PR) was located in the RabE structures and ODEs for membrane association and dissociation reactions were created in COPASI (Tables 2 and 3). Dissociation was promoted at pH below 6 and association was favored at higher pH. Fig 7a shows that the pH in RabE was almost neutral (6.7). Hence, the concentration of free HexA in these organelles was low (Fig. 7b). When the system was allowed to evolve for several minutes, the free enzyme accumulated almost exclusively in RabD organelles (Fig. 7b and c). The M6PR was given affinity only for RabE domains and remained during the simulation mostly in RabE structures (Fig. 7d), but in small amounts, it was found in RabB and C structures, and from there it leaked to the plasma membrane (Fig. 7e, recycled).

**Fig. 8:**
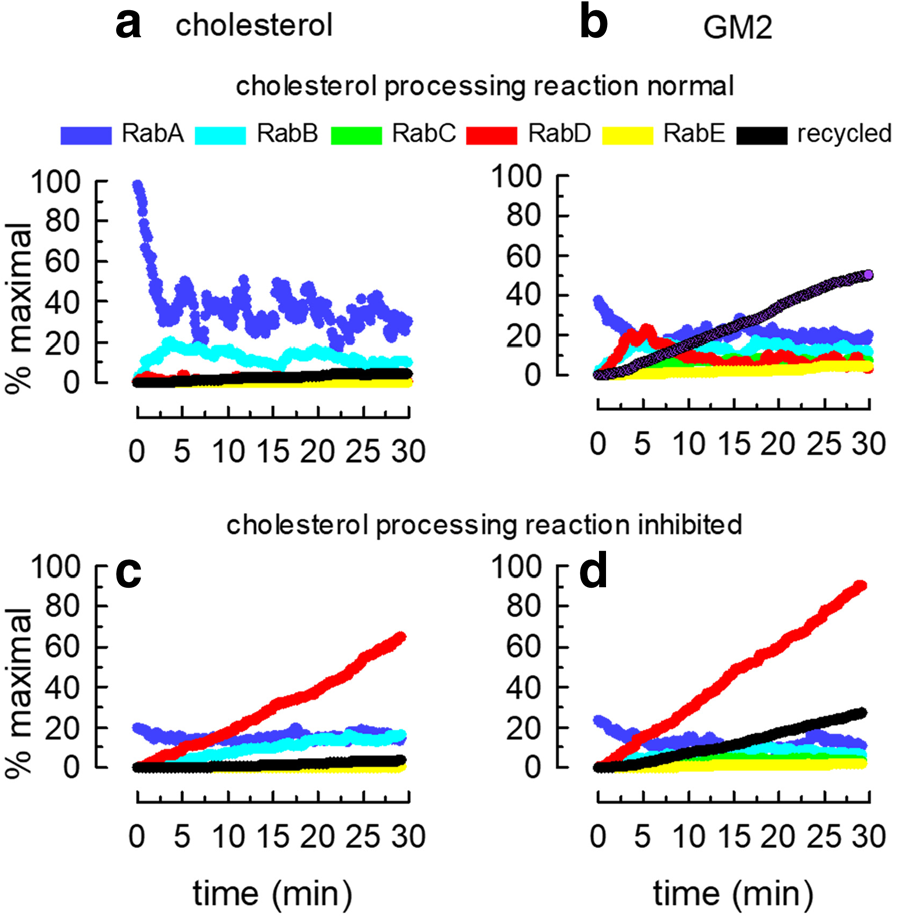
Modeling the lysosomal hydrolysis of a glycolipid. Two lipids (cholesterol-like and GM2-like molecules) were included as membrane-associated cargoes in the simulation. The lipids were loaded in newly formed RabA endosomes, mimicking the uptake from a plasma membrane domain enriched in these molecules. Cholesterol was given affinity for RabA and RabB domains and GM2 for RabD structures. Cholesterol was processed by a reaction programmed in COPASI that depends on the presence of RabD domains. GM2 was digested by a reaction programmed in COPASI that requires HexA, low pH, and low cholesterol. **a** Cholesterol distribution among different Rab domains along the simulation. Notice that after a few minutes, the lipid formed a gradient in the endocytic route with decreasing concentrations from RabA to RabD structures. **b** Same as in **a**, but for GM2. After a transient increase in RabD endosomes, GM2 was maintained at low concentrations in these late endosome/lysosomal structures. Like for cholesterol, a gradient was formed, decreasing from RabA to RabD endosomes. Notice, however, that part of the lipid recycled back to the surface. **c** When the cholesterol was not removed from RabD structures (the reaction was inhibited in COPASI), the lipid accumulated in RabD endosomes. **d** GM2 hydrolysis was inhibited by the high cholesterol levels in RabD endosomes; hence, the ganglioside content increased with time in late endosomes. Data on panel **a** to **d** correspond to the average of 10 independent simulations

Another property of the endocytic route is a differential distribution of lipids. Cholesterol strongly influences the biophysical and functional properties of membranes and modulates the activity of receptors and enzymes [32]. This lipid is present in a gradient along endosomal structures. The content of cholesterol is higher in the plasma membrane and decreases from early to late endosomes. Lysosomes have a central role in the sorting of cholesterol out of the endocytic route [33, 34]. To mimic this features of the endocytic pathway, cholesterol was included as a membrane cargo in the newly formed RabA endosomes and the cholesterol of lysosomes was decreased by a reaction modeled in COPASI (Table 3). Cholesterol was given affinity for RabA (1.0) and RabB (0.5) organelles (Table 2). Under these conditions, a cholesterol gradient was spontaneously generated after a few modeling minutes (Fig. 8a).

Acid hydrolases in the lumen of late endosomes/lysosomes are responsible for the digestion of many organic molecules, including complex lipids. Defects in this process intoxicate the cell and lead to several storage diseases. Lipid metabolism is influenced by the pH, the specific set of acid hydrolases and also by the lipid composition of the membranes [35, 36]. For example, cholesterol inhibits the activity of several lipid hydrolases [36]. With the incorporation of a lysosomal enzyme (HexA), and the regulation of pH and cholesterol content, the lysosomal digestion of a complex lipid, such as the GM2 ganglioside could be simulated. With this aim, a digestion reaction, stimulated by HexA and acidic pH, and inhibited by cholesterol was programmed in COPASI (Table 3 and Additional file 7). GM2 was included in RabA endosomes as a membrane-associated cargo with affinity for RabD domains (Table 2). The continuous uptake of the glycolipid did not lead to lysosomal accumulation and a gradient was formed with recycling of undigested GM2 to the plasma membrane (Fig. 8b). Perturbation of the conditions for glycolipid digestion (alterations in pH, HexA delivery or lysosomal cholesterol content), strongly affects the efficient digestion of GM2. For example, if the lysosomal processing of cholesterol was inhibited in COPASI, RabD structures became enriched in this lipid (Fig. 8c). High levels of cholesterol in lysosomes caused a defect in GM2 metabolism and the lipid rapidly accumulated in RabD structures (Fig. 8d).

**Fig. 9:**
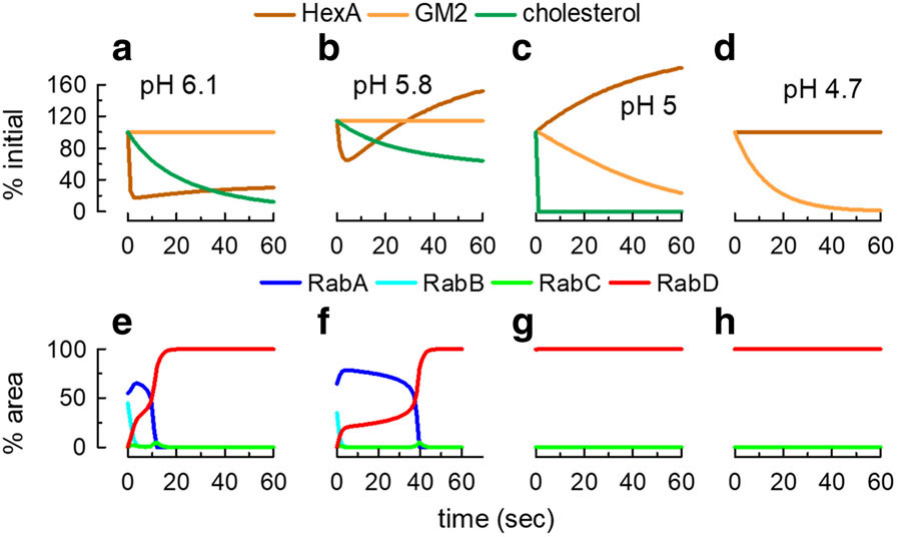
Lipid and Rab dynamics in individual endosomes. Repast stored the time series of all the network of reactions (including lipid metabolism, HexA release, and Rab dynamics) calculated in COPASI for each endosome (Additional file 3). Four endosomes were selected with different initial pH. **a**-**d** Changes in HexA and lipid content of each endosome calculated by COPASI using the initial values sent by Repast. The concentrations were expressed as a percentage of the initial value. **e**-**h**. Changes in the Rab content calculated by COPASI using the initial values sent by Repast. The values are expressed as a percentage of the total area of the endosome. Data coming from 4 individual endosomes

Conditions for GM2 hydrolysis were different in each endosome. The amount of lipid and hydrolytic enzyme, the pH and cholesterol content were peculiar for each organelle and changed with time. The simulation allowed to analyze by COPASI the glycolipid metabolism under the prevailing conditions of individual endosomes. Simultaneously, the Rab dynamics for the endosome can be followed along time. As examples, the lipid metabolism and Rab dynamics for four different endosomes are shown in Fig. 9. The first two endosomes acquired RabD characteristics (Fig. 9e and f), but the pH, cholesterol content and hydrolytic enzyme were not suitable for GM2 hydrolysis (Fig. 9a and b). On the contrary, the other two endosomes were rich in RabD (Fig. 9g and h) and digested the lipid but with different kinetics (Fig. 9c and d).

## Discussion

We present the first formal model of a complete endocytic pathway mimicking uptake, recycling, and digestion of soluble and membrane-bound cargoes. The model can be expanded and complexity can be added to deal with other specific aspects of the pathway not included in the present version. In addition, this modeling strategy is suitable to study molecular interaction and chemical transformations occurring inside endosomes where the conditions change abruptly upon fusion and fission events. These possibilities were exemplified by adding an extra TGN-like domain in the ABM model and ODE to deal with endosomal acidification, lysosomal enzyme delivery, cholesterol processing, and glycolipid digestion. Notice that the model can be expanded to deal with additional structural and biochemical complexity. It is known that intraluminal vesicles and acidic lipids are important for GM2 digestion [35, 36]. Internal vesicles can be promoted to agents carrying specific lipids and proteins, and the network of acid hydrolases and co-activators necessary for glycolipid digestion can be included in the simulation. Also, the Golgi-endosome trafficking can be completed by adding more transport molecules for the enzymes and new membrane domains to capture the complexity of this particular transport step [12, 31]. The platform is also suitable to model processes occurring in the cytosolic face of the membranes. An elongated world as shown in Fig. 2f could be a good scenario to study molecular mechanisms of organelle movement along microtubules.

The model is also a proposal about the general principles governing endosomal recycling and lysosomal targeting. The pathway modeled requires a fine tuning between maturation, which transports in bulk soluble and membrane components to late endosomes, and fusion-fission events, able to sort out membrane factors from the maturing process and direct them to other cellular destinations, including the recycle to the cell surface. From this description, it is clear that transport requires “maturation” and “vesicular transport” steps, suggesting that both mechanisms are necessary for efficient intracellular trafficking.

In the context of fusion/fission-mediated transport, movement of membrane cargoes between endosomes having different Rab domains depends on the fusion probability between the domains and partitioning preference of the cargo when the budding of vesicles or tubules segregates the membrane domains in separate structures. The idea that endosomal sorting to other cellular destinations is necessary to avoid transport to lysosomes has been proposed as informal models several times [16, 37]. We expect that having the possibility of modeling cargoes that are directed to specific membrane domains will provide a convenient platform to put together the different sorting mechanisms already described.

The random and iterative nature of fusion/fission events generates very heterogeneous sets of endosomes carrying the same Rab domain. These differences were not stable, and changed rapidly along time as new fusion and fissions occurred. Thus we conclude that endosomes with a very specific combination of conditions that might be necessary for some important events are likely to occur, with a given probability, but will not be stable and recognizable as an independent compartment.

The model also stresses the importance of low-frequency events. Without the low but significant possibility of RabB/RabD fusion, the late endosome compartment would be an irreversible sink, without any chance of rescuing material miss-sorted to this compartment by maturation. Also, luminal vesicles, capable of being recycled to the extracellular medium (exosome release-like process), and the presence of M6PR at the plasma membrane occurred by a combination of rear events.

Other formal models for intracellular transport have been previously published, in most cases, as proof of principle of different transport mechanisms [38–40]. Most of the published models use ODE. A seminal example is the paper by Heinrich and Rapoport where they showed that stable non-identical compartments can be formed by a combination of SNARE-dependent fusion and coat-dependent fission [41]. ABM has been used to model the spatial and temporal dynamics of autophagic organelles [42], and the cellular network of mitochondria [43]. We have previously applied ABM to show that soluble cargoes can be efficiently transported by endosome fusion and asymmetric fission [10]. The molecular crowding effect and the influence of the cytoskeleton on intracellular transport have been studied using a more mechanistic version of ABM [44, 45]. A combination of ABM and ODE has been used to study mitophagy [46]. However, our model is the first to simulate a complete and stable endocytic route capable of handling the uptake, recycling, and digestion of membrane-bound and soluble molecules.

The modeling platform introduced has some shortcuts, for example: i) The scenario is at present a projection in two dimensions of the real 3D space. Repast has the possibility of modeling a three dimension space, but agents and rules would have to be adapted to a more complex and computationally expensive situation. ii) The modeled space is a rectangle, which is convenient to represent a section of a cell. However, some simulations may require specific shapes that will require to generate more complex borders. iii) At present, the form of organelles depends only on their volume and area, which are not enough to represent the shape of real organelles. iv) COPASI calculations assume a homogeneous distribution of the species, including membrane-associated molecules, which may be not convenient for some situations, especially for large structures. v) Agents at a given time cannot be stored to be used for new runs. It would be desirable to let the system reach a steady state and use this set of agents to start the simulations. We expect to solve this limitation in the near future. In brief, the present version of the platform provides a convenient modeling frame for intracellular transport, and its application to concrete trafficking problems will be important to show what limitations need to be addressed in new versions.

## Conclusions

Modeling chemical reactions and molecular interaction occurring in the interior or the boundary of dynamic organelles constantly change position and composition is challenging. Our simulation relies on two complementary modeling strategies: ABM and ODE. Tools for modeling using ODE are freely available and are relatively user-friendly [2, 47]. Networks of molecular interactions, or signaling cascades can be easily modeled in COPASI. On the other hand, ABM software is ideal to model movement and interactions among organelles, including fusion and fission events. However, a drawback for ABM is that, because of its great flexibility, it is too generic to be used without some basic programming skills [48]. The connectivity between the two strategies is simple although it requires to be adjusted for different applications [23]. In the near future, we will work in a more user-friendly platform easily expandable to incorporate more domains and networks of proteins and lipids to model specific aspects of the endocytic pathway.

Modeling a particular intracellular transport process will require: i) to select the set of interacting organelles to be included, and define their properties (Rab domains, fusion compatibility, maturation, tubule tendency, migration on MT, recycling probability), ii) to select the set of molecules required for the process and define their affinity for Rab domains and their network of molecular interactions, and iii) to specify a starting set of organelles. For the GM2 hydrolysis simulation, organelles, molecules, and parameters are listed in Tables 1, 2, 3, and Additional file 1. Molecular interactions were programmed in COPASI (Additional files 5 and 7). In a first approach, parameters can be selected by educated guesses taking into account what is known about the process. Experimental values for specific steps or global behavior of the system can be used to adjust the model and narrow the parameter uncertainty.

We expect that our approach will contribute to the development of quantitative and formal hypotheses that will foster a better understanding of the highly complex and dynamic nature of the intracellular transport.

## Methods

### Agent-based model (Repast)

The freely available modeling platform Repast [48] was used to model agents and actions in an Eclipse environment (https://repast.github.io/). The code can be accessed from the Git repository https://github.com/ihem-institute/immunity/tree/LipidMetabolism

### Ordinary differential equations (COPASI)

ODEs were programmed in COPASI [2] (http://copasi.org/). All COPASI files are included in the Git repository. COPASI and Repast interaction is achieved through the RabConvertion and LipidMetabolism classes. Basically, Repast sends initial concentrations present in each endosome to COPASI that generates a time series. A matrix with time series for each metabolite is sent back to Repast.

### World

The space represented is a projection in 2D of a cytosol square of 4.5 × 4.5 μm. The size of the world can be changed using a scale factor (Fig. 2d and e). The shape of the world can also be changed, adjusting Repast parameters (Fig. 2f). The upper border corresponds to the plasma membrane and the lower border to the nucleus. The right and left borders form a continuous. Hence, the world shape corresponds to the surface of a cylinder.

### Time

The tick duration was calibrated with the fastest process in the model (movement of organelles on MT; 1 tick = 0.06 s). The time for all other actions was adjusted changing the probability of being performed at each tick. Hence, actions (see below) occurred with the following frequencies every 0.06 s: Move, 1; Fusion, 0.001; Tethering, 0.06; Internal Vesicle, 0.012; New Endosomes, 0.001; Recycling, 0.06; Lysosomal digestion, 0.006; Fission, 0.15.

### Agents

Each “Endosome” in the model has area and volume, Rab content, membrane content, and soluble content. Contents were expressed in mM units. About 20 molecules of a soluble molecule at 1 mM concentration will be present in a 20 nm radius vesicle. This value fits well with the reported range of membrane proteins in an average synaptic vesicle of 21 nm radius (2–70 molecules, [49]). Assuming that membrane molecules are in a homogeneous mix with soluble components is one of the limitations of the model.

The shape and size of endosomes correspond to a spheroid (prolate spheroid) with the area and volume of the endosome. The spheroids were represented as ellipses in the 2D representation of the world. Endosomes can perform the following actions:

### Move

The endosomes move randomly according to their angular moment calculated from their shape and volume. Basically, large organelles move and change direction more slowly than small ones. Endosomes near the plasma membrane or the nucleus move always randomly. Organelles away from the borders and near a MT (light blue straight lines in the model) may move to the plus (plasma membrane) or minus (nucleus) end of the filament. Supported by experimental evidence, the speed of movement on MT was set to 1 μm/sec for all organelles, irrespective of their size, shape or membrane domains [50]. On MT, round and tubular organelles move to the surface or to the center of the cell according to their membrane domains. Tubules move to plasma membrane with a probability proportional to the area covered by RabA, B and C. Non-tubular structures move to the cell center with a probability proportional to the area covered by RabC, D and E. In summary, tubules move to the plasma membrane if they are rich in RabA, B or C; otherwise, they move randomly. Non-tubules move to the nucleus if they are rich in RabC, D and E; otherwise, they move randomly.

### Fusion

Each endosome sensed all other endosomes at a distance less than its size (the radius of a sphere with the endosome’s volume). If nearby endosomes carry a compatible membrane domain, they fuse. Endosome compatibility was calculated as previously described [10]. In essence, the probability of fusion of single domain endosomes is specified in Table 1. For endosomes carrying more than one domain, the probability was adjusted according to the proportional area occupied by each Rab. Fused endosomes formed a single organelle carrying the area and volume and all the membrane and luminal (soluble) components of the original endosomes.

### Tethering

As a step prior to fusion, nearby endosomes carrying compatible domains can form a cluster with a high probability of moving together. The rules to form a cluster were the same than for fusion and depended on the distance among endosomes and Rab domain compatibility.

### Fission

Elongated endosomes have enough membrane to generate tubules. Long tubules could also divide into two tubules. Fission always generate tubules with a 20 nm radius carrying a single Rab domain. The Rab domain that was incorporated in the tubule was selected at random, taking into account the tubule forming probability of each Rab domain (specified in Table 1). Notice that RabD tubules grow from endosomes that have no other domains. The size of the tubule depends on the available surface of the Rab domain selected and on the endosome surface (the remaining surface should be enough to contain the volume not incorporated in the tubule). The smallest tubule formed was 40 nm long (almost a spherical vesicle). Soluble content distributed proportional to the volume of the formed structures, except luminal vesicles that could not enter into tubules. Membrane components were directed to the tubule only if they have more affinity for the Rab domain forming the tubule than for the Rab domains remaining. Since the content of membrane-associated cargoes was in area units, the budding tubules carried, at most, the cargoes corresponding to the tubule area. If the affinity for the tubule was the same than for the rest of the organelle, the cargo distributed proportionally to the area of the two new organelles. The area, volume, Rab, membrane, and soluble contents were preserved during fusion and fission events.

### Internal vesicles

Endosomes carrying RabA, B or D domains could form internal vesicles (20 nm radius sphere). The area of the organelle decreased (the surface of the sphere was subtracted) and the volume increased (the volume of the sphere was added). The soluble content was not affected, but membrane content decreased proportionally to the internalized area. The internal vesicle was considered as a soluble content. The number of internal vesicles of each endosome was displayed as an integer number at the center of the organelles (Figs. 1a and 2).

### New endosomes

New endosomes were allowed to form. Rab domains were conserved during fusion and fission, but not during the dynamics of Rab domains controlled by COPASI. The more dramatic change was the maturation of RabA to RabD. Hence, the total amount of Rab domains in the system was assessed and differences with the initial values were compensated by the formation of new endosomes. By large, most new endosomes carried RabA domains to compensate for maturation. When RabA organelles were formed, they were modeled as an endocytosis, budding from the cell surface and carrying soluble and membrane-bound components present at the plasma membrane.

### Recycling

When endosomes carrying RabA or RabC membrane domains were proximal to the plasma membrane (less than the organelle’s radius), they could release their membrane and soluble content (recycled material), that was incorporated into the plasma membrane. Not all cargoes could recycle from RabA endosomes. For example, MHC-I only recycled from RabC endosomes.

### Lysosomal digestion

Endosomes carrying RabD domains decreased their volume by a 0.1%; as a consequence, the extra surface available was invaginated as internal vesicles. In this way, the RabD area decreased. This process compensates for the continuous generation of RabD domains caused by the early-to-late endosomal maturation. Lysosomal digestion also consumed 0.1% of the membrane and soluble cargoes. The number of internal vesicles decreased by one with a probability calculated as 0.01 times the number of internal vesicles in the endosome.

### Microtubules

Straight lines were drawn in the model representing MT. In the present model, MT can only change position with a 0.0001 probability and are used to direct the movement of endosomes.

### Plasma membrane

It is a single and static agent. This agent contains soluble and membrane molecules. Plasma membrane accumulates the material recycled, and according to the process modeled, its composition was used to generate new early endosomes.

### Rab dynamics – COPASI

ODEs were programmed with COPASI to simulate Rab dynamics (Additional file 5). The reactions modeled are represented in Fig. 1c. Basically, all domains stimulate their own activation and blocked their inactivation. In addition, RabA stimulates RabD activation and inhibits its inactivation whereas RabD inhibits RabA activation and stimulates its inactivation (Fig. 1c). A cut-out switch has been previously described for Rab5/Rab7 conversion [22]. Rabs could be released to the cytosol and re-associate with membranes. The reactions, kinetic functions, and parameters used are shown in Table 3. COPASI was called from Repast for each endosome providing the initial concentrations of each species. For membrane components, the concentration was assigned as the proportion of the endosome surface in mM units (e.g., Rab concentration was 1 mM for an endosome completely covered by a single Rab domain). This value was estimated from the reported number of 10 Rab3A molecules present in an average synaptic vesicle of 21 nm radius [49]. COPASI generated a time series for 50 s (833 ticks). The time series was loaded in each endosome and Repast actualized the values of the species using the stored values. The time series was lost and COPASI had to be called again whenever an action modified the content of the endosome (e.g., fusion, fission, recycling).

### Lipid metabolism – COPASI

To model the lysosomal digestion of GM2, new molecules and reactions were added to the simulation (see details in Tables 2 and 3). First, early endosomes were loaded with a vATPase with affinity for Rab A and RabD domains, cholesterol with affinity for RabA and B domains, and GM2 with affinity for RabD domains. RabE structures were included in the simulations carrying M6PR as a membrane cargo and HexA bound to the carrier. M6PR has affinity for RabE domains. RabE structures fuse with other Rab domains with probabilities specified in Table 1 (the largest value was 0.01 and corresponds to fusion with RabB domains). COPASI was used to generate the ODEs for endosome acidification, HexA association/dissociation (it was considered a soluble cargo when dissociated), cholesterol processing, and glycolipid digestion (Additional file 7). The reactions, kinetic functions, and parameters used are shown in Table 3.

### Markers

Soluble and membrane-bound markers were included when required. They were modeled as soluble or membrane-bound cargoes, with the special characteristic that they were not digested and that they could not be fractionated. Hence, during fission, they were allocated in one of the two new organelles with a probability that was proportional to the partition properties of the marker. For example, if the marker was soluble, it went to one or the other structure with a probability that depends on the relative volume of both newly formed endosomes.

### Model initialization

The parameters and initial organelle characteristics were loaded from a csv (comma-separated values) file generated from a spreadsheet (Additional file 1). The parameters could be changed by altering the csv file during the simulation. All COPASI files were pre-loaded in the Eclipse environment to be called from Repast when needed.

### Transport of markers

The simulation generated a csv file with the characteristics of the endosomes carrying markers (Rab, soluble and membrane content, area and volume of the organelle). The data was collected every 100 ticks (Additional file 2). These values were used in Figs. 3 and 6b.

### Membrane and soluble cargo distribution

The simulation calculated the amount of each soluble and membrane cargoes associated with the different Rab domains. For example, to estimate the amount of internal vesicles associated with RabD domains, the number of internal vesicles present in each endosome was multiplied by the relative content of RabD on the organelle (number of internal vesicles * RabD area/total endosome area) and added to a total sum (Additional file 2). The same calculation was done for soluble or membrane-bound cargoes. As a rule, the simulation was run several times and the values plotted in the figures are the average of 5–10 runs.

### Snapshots

The complete set of agents (e.g., endosomes) at a given time could be listed using a Repast routine (Additional file 3). From there, histograms could be generated by calculating the frequency distribution of specific values, such as pH, number of internal vesicles, etc. As a rule, the endosomes of several (4–8) simulation at a given time were combined to calculate the histograms.

## Reviewers’ comments

### Reviewer’s report 1: Rafael Fernández-Chacón, Instituto de Biomedicina de Sevilla (IBiS), Spain

This is an interesting work focused on the development of a novel and original in silico tool to model the dynamics of membrane trafficking and biogenesis of endosomes. The model have attempted to include an important number of biological variables with a special emphasis in the fate and behavior of organelles depending on the proportion of different Rab proteins on their membrane. To model membrane trafficking is certainly challenging but, on the other hand, of enormous potential to gain quantitative insight about the molecular details of different fusion/fission events, organelle transport and cargo transfer and delivery. The tool seems to be open source and the optimization to make it more user friendly is in progress. My main concern is about the connection to actual biological data.

Author’s response: *Thank you for pointing out the importance of modeling intracellular transport. We understand your concern. We want to highlight the flexibility of the platform, wherein a qualitative idea about how a transport process works may be sufficient to generate a model. However, each step can be upgraded to include a precise molecular mechanism. Hence, experimental manipulations of a specific molecular target can be introduced in the model, and the effect on the simulation compared with experimental results.*

1. On one hand it is not clear in the manuscript how values such as probability of homotypic fusion or maturation have been inferred from biological observations. For other phenomena such as synaptic transmission some of the parameters (for example probability of release) are easier to quantify and therefore easier to be transferred to models. I think it would be useful to provide a table presenting the key biological parameters used in the model and succinctly explain the biological rationale behind, ideally accompanied by relevant bibliography.

Author’s response: *Following the reviewer’s advice, the main parameters used in the model are included in three separate tables, each one with a short rationale about the values. We added some pertinent references for parameter estimation such as the number of molecules per organelle.*

(2) On the other hand it would also be useful to illustrate any biological prediction, even a simple one, that the model could make. Would it be possible to compare results obtained with model with real observations from a well defined biological situation in which any of the proteins involved have been somehow interfered either genetically or with a pharmacological strategy?

Author’s response: *Following the reviewer’s advice we simulated a RabD (late endosome) knockdown. It is known that Rab7 is one of the main Rabs responsible for lysosomal biogenesis. The new* Fig. 5h *shows that transport of a soluble cargo was impaired and, unexpectedly, large early/sorting endosomes were observed. Similar effects have been reported in Rab7 knockdown cells.*

(3) In order to smooth the path for users willing to apply the model to the analysis or simulation of biological situations, would it be possible to provide a list of parameters suitable to be measured with the cell biological approaches that could be used as inputs in the model?

Author’s response: *Kinetics of cargo transport to different intracellular compartments or recycling to the plasma membrane are relatively easy to find in the bibliography or to measure in the laboratory. These kinetics can be used to set several properties of the model (organelles involved, parameters, etc).*

### Reviewer’s report 2: James Faeder, Los Alamos National Laboratory, USA

This paper presents a modeling framework and simulations results for a hybrid agent-based approach to simulating endocytosis in mammalian cells. The scope and detail of the model presented are substantially greater than previous efforts, and this framework offers the potential for comprehensive modeling of signaling and metabolic processes coupled with endocytosis. Thus, the modeling framework presented here represents a substantial advance over the current state-of-the-art and may be useful to both computational modelers and cell biologists. The modeling approach is based on using an agent-based simulator (REPAST) to model the plasma membrane and endosomes as agents with spatial attributes coupled to an ODE simulator (Copasi) to simulate biochemical reactions within agents. The general nature of both software frameworks employed and the fact that they are freely-available and open-source, makes this an attractive approach on which to build future efforts. The model is validated in a qualitative fashion through the consideration of a number of different scenarios, including pulse-chase experiments tracking various endocytic cargoes, endoscope acidification, trafficking of lysosomal enzymes, and lipid trafficking. The paper does have a number of shortcomings that I think it would be useful for the authors to address:

Author’s response*: We thanks the reviewer for the positive remarks.*

1. Software availability and documentation. The GitHub links given in the text don’t exist when I try to find them. It seems that the files must be on the branch LipidMetabolism, but the directory structure still doesn’t match what is found in the text. I can find the Java source code presumably used to generate the simulation results shown in the paper, but there is no documentation, so it would be very difficult for anyone else to use the code productively at this point.

Author’s response: *the LipidMetabolism branch has been updated.*

2. Model validation. Many of the simulations don’t appear to reach a steady state but are terminated at 30 min or some other relatively short time period. Why is time zero here being treated as the resting state of a cell? Why are the dynamics being analyzed in the text relevant for an actual cell, which must establish a resting steady state and then respond to perturbations? Here, the dynamics being considered seem arbitrary. For Fig. 4 onward, what are the initial conditions of the simulation. Is a steady state established prior to starting the simulation. If not, why not?

Author’s response: *The simulations were started from an arbitrary initial condition (specified in* Additional file 1*). It rapidly evolved (~ 3 min, equivalent to ~ 3000 ticks) to a set of organelles that remains qualitatively stable for more than 60 min. Following the reviewer’s advice, we designed a pulse-chase protocol starting 3 min after the beginning of the simulation. We did not observe any major difference in the destination of cargoes under these conditions (*Additional file 6*). However, we agree that a better starting point would be a set of organelles at steady state. Repast has a freeze-dry function that allows to store and recover a model at any time point. Unfortunately, the freeze-dry function is not working for our model. We expect to solve the problem in a new version. The 30 min time for simulations was selected because experimentally it is known that transferrin recycling and soluble cargo targeting to lysosomes occur in this timescale.*

3. Need to mention explicitly in the main text the technical limitations: 2D, simple geometry (square), fixed time step. Also the lack of detailed validation against models is a significant concern.

Author’s response: *A complete paragraph enumerating some of the limitations of the present version of the modeling platform was added at the end of Results. We understand the concern about validation but the idea of this report was to present the platform and to show that it can be used to simulate processes that require intracellular transport, such as cargo sorting and glycolipid digestion. Validation of each assumption introduced will require the design of specific experiments.*

4. Table 2. What are the units of the various parameters? What is the source for the models included in the reactions and how were the parameters chosen?

Author’s response: *The Table is now* Table 3 *and units are described in the table’s legend. A short rationale for the parameter values was added to the Table’s legend.*

5. Table 1S could use some documentation — what is the structure of this file? Where is that explained?

Author’s response: Table 1S *is now* Additional file 1*. The documentation was included in the same Excel worksheet, as comments to appropriate cells.*

6. For the simulation results that were determined by averaging over several trajectories (Figs. 4, 5, 6, 7, 8 and 9) there should be some statement about the observed variations? Were such small numbers of repetitions sufficient to obtain converged results? Why were such a small (by simulation standards) number of replicates performed?

Author’s response: *Simulation trajectories were very consistent. Six individual runs of a pulse-chase protocol are included in* Additional file 6*. Notice that in each trajectory transferrin is recycled and dextran transported to late endosomes.*

### Reviewer’s report 3: Thomas Simmen, University of Alberta, Canada

The paper by Mayorga provides mathematic modelling of endosomal trafficking pathways following endocytosis. As a bioinformatic approach, the manuscript provides useful information, as it faithfully models the rather complex pathways taking place at the plasma membrane, tied to endosomal organelle subtypes. The models lead to astonishing reproductions of published findings on endocytic trafficking. However, the models are based on seemingly arbitrary choices of settings and should be further tested and validated as outlined in the specific points. Additional approaches may be necessary to address this concern.

Author’s response: *We thanks the reviewer for the positive remarks. We understand the reviewer’s concern. However, the choice of setting and parameters were based on the qualitative idea most of the cell biologists have about transport in the endocytic pathway. We adjusted the maturation and fusion/fission mechanisms of transport, Rab dynamics, and the domain affinities of cargoes to reproduce experimental data of transferrin recycling and early endosome maturation. The* in silico *knockout and knockdown results show that the chosen setting was able to reproduce several experimental observations. However, a sensitivity analysis would be necessary to assess the influence of the different parameters on the model behavior.*

Specific points:

1. The description of the different behaviors of the membrane versus soluble marker needs to be improved, as it is currently confusing. The statement about fission impacting membrane and soluble proteins differently should be elaborated and mentioned earlier as a hypothesis.

Author’s response: *Following the reviewer’s advice, the subject was introduced in Background.*

2. The description of the model leads to elegant reproductions of real live cells and their endocytic system, including the TGN. However, in many cases, seemingly arbitrary choices were made, for instance for the inclusion of supposed activities of proton pumps or cholesterol affinities. It would be necessary to add some “biology” to further test these systems. One way could be to test what happens with dominant-active or dominant-negative Rabs. Are the reported distributions seen?

Author’s response: *The settings to simulate vATPase and cholesterol distribution were selected to reproduce what is known about the sterol and pH gradients in the cell (pH and cholesterol concentrations decrease from early to late endosomes). Although no molecular mechanisms were included, a search in the bibliography shows that Arf6 and ARNO (two early endosome resident proteins) and RILP (a Rab7 effector) interact with subunits of the vATPase. These references were included in the text.*

3. What about the functioning of sorting signals? How can these be incorporated into the model?

Author’s response: *Sorting signals, such as short peptides present in the cytosolic portion of transmembrane proteins, play a central role in the molecular mechanisms that determine the localization of a factor in the cell. This concept is outlined in Background and Results (Transport of Soluble and Membrane-bound Cargoes). In the present model, the destination of a membrane factor is specified by its membrane domain affinity. However, molecules with tags that interact with coat factors can be included in future models. It is possible to program a membrane domain with some tag-carrying cargo, which would recruit a specific cytosolic coat protein that will foster the scission of a cargo-rich organelle.*

## Additional files

Additional file 1: Model parameters and initial organelles. Explanatory comments are included in several cells. (XLSX 18 kb)

Additional file 2: Output of the simulation I. Contains i) the organelle distribution of cargoes along time and ii) the characteristics of the organelles visited by markers along time. (XLSX 478 kb)

Additional file 3: Output of the simulation II. Contains a list of all agents in the simulation at a specific time. (XLSX 3343 kb)

Additional file 4: Movie generated by Repast. (PPTX 18029 kb)

Additional file 5 COPASI file (Rab dynamics). Should be run with COPASI (http://copasi.org/). (CPS 130 kb)

Additional file 6: Pulse-chase simulations. (DOCX 211 kb)

Additional file 7: COPASI file (Lipid Metabolism). Should be run with COPASI (http://copasi.org/). (CPS 67 kb)

## Abbreviations

ABM: Agent-based model
COPASI: COmplex PAthway Simulator
GM2: A monosialic ganglioside
HexA: β-hexosaminidase A
M6PR: Mannose 6-phosphate receptor
MHC-I: Major histocompatibility complex class I
MT: Microtubule/Microtubules
Tf: Transferrin
TGN: trans-Golgi network
vATPase: Vacuolar proton pump ATPase
