# Supplementary figures and images for: Reconstruction of endosomal organization and function by a combination of ODE and agent-based modeling strategies
Source: Biol Direct. 2018 Nov 23;13:25. doi: 10.1186/s13062-018-0227-4 (PMC6883406; doi:10.1186/s13062-018-0227-4)

## Slide 1
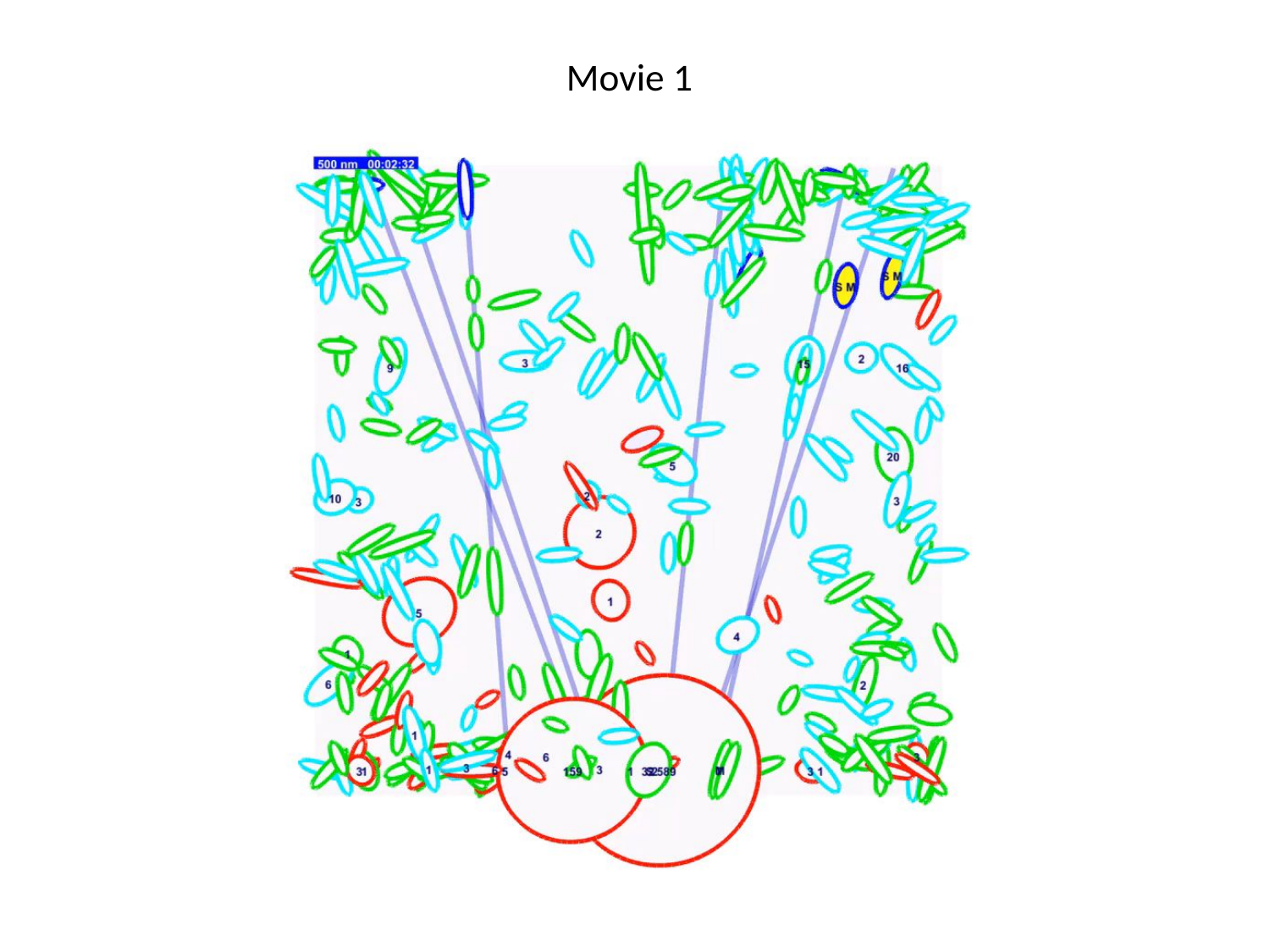

Movie 1

Supplement: Supplementary file 4 — Movie generated by Repast. (PPTX 18029 kb) [file 13062_2018_227_MOESM4_ESM.pptx]
