# Supplementary material for: Reconstruction of endosomal organization and function by a combination of ODE and agent-based modeling strategies
Source: Biol Direct. 2018 Nov 23;13:25. doi: 10.1186/s13062-018-0227-4 (PMC6883406; doi:10.1186/s13062-018-0227-4)
Supplement: Supplementary file 6 — Pulse-chase simulations. (DOCX 211 kb) [file 13062_2018_227_MOESM6_ESM.docx]

**Pulse/chase of a soluble (dextran-like) and a membrane-bound (Tf-like) cargoes.** RabA endosomes were empty at the beginning of the simulation. Uptake was allowed in the 3-6 min time period. A and B. Association of cargoes with different Rab domains and recycling to the plasma membrane during different chase times. Notice that the membrane markers efficiently recycled to the plasma membrane whereas the soluble marker accumulated in RabD endosomes. The values for the large figures at the left correspond to the average of the six individual simulation shown at the right.
